# Change in Work-Related Income Following the Uptake of Treatment for Mental Disorders Among Young Migrant and Non-migrant Women in Norway: A National Register Study

**DOI:** 10.3389/fpubh.2021.736624

**Published:** 2022-01-07

**Authors:** Kamila Angelika Hynek, Anna-Clara Hollander, Aart C. Liefbroer, Lars Johan Hauge, Melanie Lindsay Straiton

**Affiliations:** ^1^Department of Mental Health and Suicide, Norwegian Institute of Public Health, Oslo, Norway; ^2^Faculty of Health Sciences, Oslo Metropolitan University, Oslo, Norway; ^3^Department of Global Public Health, Karolinksa Institutet, Stockholm, Sweden; ^4^Netherlands Interdisciplinary Demographic Institute, The Hague, Netherlands; ^5^Department of Epidemiology, University Medical Centre Groningen, University of Groningen, Groningen, Netherlands; ^6^Department of Sociology, Vrije Universiteit Amsterdam, Amsterdam, Netherlands

**Keywords:** early adulthood, income, mental disorder, migrant women, national register data, outpatient mental health care

## Abstract

**Background:** Women, and migrant women in particular, are at increased risk of many common mental disorders, which may potentially impact their labor market participation and their work-related income. Previous research found that mental disorders are associated with several work-related outcomes such as loss of income, however, not much is known about how this varies with migrant background. This study investigated the change in work-related income following the uptake of outpatient mental healthcare (OPMH) treatment, a proxy for mental disorder, in young women with and without migrant background. Additionally, we looked at how the association varied by income level.

**Methods:** Using data from four national registries, the study population consisted of women aged 23–40 years residing in Norway for at least three consecutive years between 2006 and 2013 (N = 640,527). By using a stratified linear regression with individual fixed effects, we investigated differences between majority women, descendants and eight migrant groups. Interaction analysis was conducted in order to examine differences in income loss following the uptake of OPMH treatment among women with and without migrant background.

**Results:** Results showed that OPMH treatment was associated with a decrease in income for all groups. However, the negative effect was stronger among those with low income. Only migrant women from Western and EU Eastern Europe with a high income were not significantly affected following OPMH treatment.

**Conclusion:** Experiencing a mental disorder during a critical age for establishment in the labor market can negatively affect not only income, but also future workforce participation, and increase dependency on social welfare services and other health outcomes, regardless of migrant background. Loss of income due to mental disorders can also affect future mental health, resulting in a vicious circle and contributing to more inequalities in the society.

## Introduction

Mental disorders are one of the main reasons for labor market marginalization due to significant impairment of daily functioning and the ability to participate in the labor market (1). Mental disorders are linked to increased unemployment, loss of work-related productivity and income (2, 3). In turn, this can increase dependency on social welfare services, resulting in large economic costs for the whole society (4). Mental disorders are most prevalent during early adulthood due to the multiple critical changes young adults experience, such as labor-market establishment (5). Women are particularly at risk (6, 7).

Furthermore, migrants, especially women, are at increased risk of many, mental disorders such as depression, anxiety, or posttraumatic stress disorder, when compared to the majority population (8–10). Differences in the risk of mental disorder are found by country and region of origin (11). Several register-based studies from Norway and Sweden investigated differences in mental health between majority and migrants. A general pattern emerging from these studies is that migrants originating from Middle East and North Africa and former Yugoslavia are among those with the highest odds of mental disorder, whereas migrants from EU Eastern Europe, Sub-Saharan Africa, and Asia have lower odds (12–15). Yet, despite evidence of increased vulnerability of developing mental disorders among some migrant groups, several studies suggest that some use mental healthcare services to a lesser extent than the non-migrant population, with differences by country of origin and by reason for migration (14, 16, 17). In general, refugees are found to have higher mental healthcare service use than non-refugees and non-migrant population (17), while labor migrants to have lower use (16). Thus, migrant status is not only a risk factor for mental disorder but also for an unmet need for treatment (18).

In addition to an increased risk of mental disorders, women have lower employment rates than men (19), and the gender gap in wages in many countries is still large (20). The gender difference in employment rates is even larger for migrants. Migrant women, particularly women from Africa and Asia, have far lower employment rates than both non-migrants and migrant men (21). Furthermore, migrant women are often overrepresented in lower-skilled jobs (22), however, as in the case of mental disorders, large differences are found by region of origin (21), and migrants from Africa and Asia seem to be the most disadvantaged (23, 24). Women from Africa and Asia have the lowest employment rates in Norwegian society (about 50%), whereas among women from European countries, employment rates are much higher (above 70%) (21). The differences in employment between different migrant groups may be attributed to their reasons for migration, but also to differences in educational attainment and whether qualifications of the women fit the Norwegian labor market. Migrants from Africa and Asia including Turkey come mainly to Norway through family reunification or as refugees (25), and a large share of those women have compulsory education only (26). Among migrants from the EU countries, the vast majority are either labor or family reunified migrants (25) and more than half of all women from the EU have higher education (26). Additionally, poor language proficiency, and low competence or lack of required experience can also influence labor market participation for many migrants (27).

Compared with men, women generally have lower rates of labor market participation (19), lower income levels (20) and are at greater risk of many common mental disorders (6, 7). Further, evidence suggests that poor mental health may have a more negative effect on women's labor market participation than on men's (1). Thus, this study focuses on women. Furthermore, given the disadvantage that migrant women face, both in terms of increased risk of mental disorders and poorer labor market attachment, compared with majority women, it is of importance to study whether mental disorders may affect labor market outcomes differently for migrant and non-migrant women.

The association between mental disorders and labor market participation may be due to poor labor market attachment causing mental disorder (causation) or by mental disorder causing adverse work-related outcomes (selection) (28). There is support for both perspectives and evidence suggests they are not mutually exclusive. In this study, we use longitudinal data to examine the selection perspective, by examining income differences between people who have and who have not been treated for mental health issues. The selection perspective assumes that experiencing a mental disorder can hinder socioeconomic development. Mental disorders during early adulthood can impede socioeconomic development by affecting daily functioning and the ability to actively participate in the labor market, as well as in society at large (1, 29, 30), thus influencing labor market participation and income.

Previous studies have found that mental disorders have a negative impact on several work-related outcomes such as poor, or lack of, labor market attachment (31–35) and lower earnings, with an income reduction of between 6.5 and 20.0 percent (36–39). This loss of income has been explained by, for instance, reduced productivity or concentration. However, the effect of mental disorders on income varies by type of disorder and across the income distribution. The most adverse effect is seen in the lowest distribution tail (38, 39).

Studies focusing on differences between migrants and non-migrants found that mental disorders, defined by use of in- and outpatient mental healthcare services, increase the risk of labor market marginalization including disability pension, sickness absence and unemployment in both non-migrants and migrants (40–42). Swedish-born individuals with mental disorders had a higher risk of both disability pension and sickness absence when compared to Western and non-Western migrants, but lower risk of unemployment, especially compared to non-Western migrants (41). These differences were mainly explained by migrants' poorer previous labor market attachment, with many not qualifying for, or having limited access to, sickness and disability benefits. Furthermore, migrants with common mental disorders had higher levels of long-term unemployment when compared to non-migrants with common mental disorders (40). Despite the increased focus on the association between mental disorders and work-related outcomes, the focus on differences by gender or migrant background is still limited (43). Migrant women, especially those from non-Western countries, are at increased risk of both mental disorders (8), and poor labor market attachment and lower earnings compared with non-migrant women (21). Thus, if having a mental disorder impacts earning capacity, it is possible that migrant women may face a triple disadvantage due to being a woman, a migrant and having a mental disorder. This makes their situation interesting and important to investigate whether the effect of mental disorders on work-related income differs for women with and without migrant background. Furthermore, as previous studies [e.g. (38, 39)] found the effect of mental disorders on earnings differs depending on the initial income level, we also consider the differences between women in low- and high-income brackets. We hypothesize that migrant women will experience greater loss of income following the treatment for mental disorders when compared to the Norwegian majority and descendant women, regardless of income group. In this study, we use the uptake of outpatient mental healthcare (OPMH) treatment as a proxy for mental disorder.

## Materials and Methods

### Data Sources

This study is a dynamic national register-based prospective cohort study. We used data from the total resident population of women in Norway. A unique de-identifiable version of a personal identification number (PIN) was used to combine the information from four national registers. PIN is assigned to all Norwegian citizens at birth, as well as to all individuals registered as residents in Norway for at least six months. The Central Population Registry provided demographic information such as birth year, country of origin, migrant background and marital status. The KUHR database contains information on compensation claims from health professionals such as those working in OPMH services. From this we were able to identify individuals with OPMH consultations during the years 2006–2013. Information on the highest obtained education level was extracted from the National Education Database, Statistics Norway provided information on work-related income and child benefits for the years 2006–2013.

### Study Design and Population

We included women aged 23–40 years, born between 1968 and 1988, who resided in Norway for at least three consecutive years during the study period 2006–2013. This age group was chosen because by age 23, the majority of women have completed their education and are entering the labor force. Women aged 18–22 were excluded as during this age, many are still in education and therefore have a limited and non-stable labor market attachment. A disruption in establishing oneself in the labor market, such as the onset of a mental disorder, can have both short-term and long-term effects. All women were followed from the start of the study in 2006, when turning 23 or from the year of migration to Norway. Censoring occurred at the end of the study in 2013, when turning 40 years or the year of emigration or death. Furthermore, we excluded women who had no work-related income throughout the study period (N = 33,755), as we were interested in the *change* in work-related income. The eligible sample consisted of 640,527 women in total.

### Measures

The outcome variable was work-related income, defined as yearly pre-tax wages and income from self-employment for the years 2006–2013. Income for all years was inflated to 2013 levels by using the Norwegian consumer price index. Negative values were recoded to 0 and values higher than 2,000,000 Norwegian kroners (NOK) were set to 2,000,000 NOK. Two million NOK corresponds to 230,035 US Dollars or 200,879 Euro (currency per 15th November, 2021). This threshold was set due to women with a higher income representing extreme outliers and accounting for only 0.04% of the sample. Furthermore, to ease the interpretation of the results, income was recalculated into percentiles and presented as percentile change in income following the uptake of OPMH treatment.

The main exposure variable was OPMH treatment, used as a proxy for mental disorder. Since the first consultation is mainly used to map whether there is a need for further OPMH follow-up, to be exposed, a woman needed to have at least two consultations within a six-month period. In cases where the two contacts occurred in two consecutive calendar years, the year of exposure was set as the year of first contact with OPMH services. Furthermore, to detect changes in income following the uptake of OPMH treatment, we introduced a washout period where each woman defined as exposed had to have at least 2 years free of OPMH consultations prior to exposure. This was to increase the probability that the contact detected between 2008 and 2013 was a new contact, and not an ongoing, case.

Additionally, we considered differences in loss of work-related income following the uptake of OPMH treatment by migrant background. Migrant background was divided into three major groups: majority (Norwegian-born with at least one Norwegian-born parent), descendant (Norwegian-born with two foreign-born parents) and migrant (foreign-born with two foreign-born parents). We divided migrants into eight regions of origin: (1) Nordics, (2) Western Europe, (3) EU Eastern Europe, (4) non-EU Eastern Europe, 5) Middle East and North Africa (MENA), (6) Sub-Saharan Africa, (7) South Asia, and (8) East/ South East Asia. Women from countries not fitting into the categories presented above (N = 9,220) were excluded from the study sample due to low numbers and lack of comparability. The Norwegian majority group was used as a reference group in all analyses.

Covariates included age, educational level, marital status and motherhood, all time variant. Age was measured as a continuous variable with values between 23 and 40. Education level was a categorical variable with categories (1) compulsory or lower, (2) upper-secondary, (3) tertiary, and (4) unknown. Marital status was also a categorical variable with values (1) unmarried, (2) married/partner, and (3) previously married. Motherhood (yes/no) was based on whether the woman was receiving child benefit or not, commonly entitled to mothers with a dependent child below the age of 18 (44). We also controlled for the year of observation to adjust for time fixed effects, with a dummy variable for each year.

### Statistical Analysis

To investigate the effect of OPMH treatment on work-related income, we applied a linear regression model with individual fixed effects. Use of fixed effects models provides a method of assessing the association between exposure (OPMH treatment) and outcome (income), adjusting for all measured time-varying and both measured and unmeasured time-invariant factors within an individual, eliminating confounding from such factors (45). Each woman is treated as her own control (46). A disadvantage of using a fixed effects model is that coefficients for time-invariant variables, such as for migrant background, are not estimated (45). However, by including an interaction term between OPMH treatment (time-variant) and migrant background (time-invariant), the fixed effects models provide coefficients for the interaction between these two variables. Due to a large difference in income between the investigated migrant groups, the analysis was stratified into low- (the bottom third of the income distribution) and high- (the top two thirds of the income distribution) income levels, based on mean work-related income during the years in the study. This distribution divide was chosen to ensure there was a sufficient number of individuals in each group. The regression analyses had a hierarchical set-up with adjustment for age and an interaction term between OPMH treatment and migrant background in Model 1 and educational level (coefficient for the individuals with unknown education were omitted from the analysis), marital status, motherhood and time fixed effects in Model 2. Results for time fixed effects are not shown. Stata 15.0 was used to perform all analyses.

## Results

Table 1 shows the characteristics of the study sample, presented by migrant background and region of origin. All measures, except for income, are presented for the last year in the study. The study sample consisted of 83.2% majority women, 0.8% descendant women and 16.0% migrant women. For a detailed distribution of migrant women by region of origin, see Table 1. OPMH treatment was most common among migrant women from MENA with 8.7%, and least common among migrant women from EU Eastern Europe and East/ South-East Asia (2.3 and 2.2% respectively). Migrant women in general had a lower work-related income, both mean and median, compared to majority and descendant women, though there were large differences by region of origin. Women from the MENA and from Sub-Saharan Africa had the lowest, while women from the Nordics and from Western Europe had the highest work-related income. For other characteristics of the study sample see Table 1.

**Table 1:** Characteristics of the study sample, by migrant background.

	**Total**	**Migrant background**					**Region of origin**					
		**Majority**	**Descendant**	**Migrant**	**Nordics**	**Western Europe**	**EU Eastern Europe**	**Non-EU Eastern Europe**	**MENA**	**Sub-Saharan Africa**	**South Asia**	**East/ South East Asia**
Number of observations	4,044,019	3,363,342	33,398	647,279	77,189	53,516	130,552	75,863	68,945	59,412	55,017	126,785
Number of individuals	640,527	522,731	5,324	112,472	13,867	9,717	24,948	12,336	10,911	9,943	8,774	21,976
% of the population		83.2	0.8	16.0	2.1	2.0	4.0	2.0	2.1	2.1	1.7	3.8
% of migrants	-	-	-	-	12.3	8.6	22.2	11.0	9.7	8.8	7.8	19.5
Years in study, mean (SD)	6.3 (1.9)	6.4 (1.8)	6.3 (1.9)	5.8 (1.9)	5.6 (2.0)	5.5 (1.8)	5.2 (1.8)	6.1 (1.9)	6.3 (1.8)	6.0 (1.8)	6.3 (1.8)	5.8 (1.9)
Age, mean (SD)	34.5 (5.1)	34.7 (5.1)	30.7 (4.5)	33.7 (4.8)	33.5 (5.1)	34.7 (4.6)	32.7 (4.6)	33.9 (4.9)	34.0 (4.9)	33.8 (4.8)	34.2 (4.9)	34.1 (4.8)
OPMH service use, N (%)												
Yes	39,605 (6.2)	34,615 (6.6)	333 (6.7)	4,657 (4.1)	762 (5.5)	415 (4.3)	575 (2.3)	654 (5.3)	952 (8.7)	370 (3.7)	448 (5.1)	481 (2.2)
No	600,922 (93.8)	488,116 (93.4)	4,991 (93.3)	107,815 (95.9)	13,105 (94.5)	9,302 (95.7)	24,373 (97.7)	11,682 (94.7)	9,959 (91.3)	9,573 (96.3)	8,326 (94.9)	21,495 (97.8)
Personal income (percentiles), mean (SD)^a^	50.4 (29.2)	53.0 (28.4)	45.5 (30.4)	37.2 (29.6)	51.7 (30.8)	48.7 (32.9)	37.2 (27.1)	40.0 (29.9)	29.9 (27.7)	27.2 (26.2)	32.1 (28.7)	33.5 (27.8)
Personal income (percentiles), Median^a^	51	54	43	32	52	46	33	35	23	19	25	27
Income group (%)^a^												
Low	33.3	28.6	41.8	57.3	34.6	40.4	57.2	52.5	69.8	73.8	65.5	63.3
High	66.7	71.4	58.2	42.7	65.4	59.6	42.8	47.5	30.3	26.2	34.5	36.7
Educational level, N (%)												
Compulsory or lower	90,570 (14.1)	65,641 (12.6)	970 (18.2)	23,959 (21.3)	842 (6.1)	752 (7.7)	2,187 (8.8)	2,457 (19.9)	4,072 (37.3)	4,338 (43.6)	3,122 (35.6)	6,189 (28.2)
Upper-secondary	195,626 (30.5)	169,884 (32.5)	1,588 (29.8)	24,154 (21.5)	3,538 (25.5)	1,250 (12.9)	5,851 (23.5)	2,906 (23.6)	2,339 (21.4)	2,179 (21.9)	1,852 (21.1)	4,239 (19.3)
Tertiary	333,383 (52.1)	286,553 (54.8)	2,693 (50.6)	44,137 (39.2)	6,528 (47.1)	5,893 (60.7)	11,002 (44.1)	5,825 (47.2)	3,044 (27.9)	2,197 (22.1)	2,428 (27.7)	7,220 (32.9)
Unknown	20,948 (3.3)	653 (0.1)	73 (1.4)	20,222 (18.0)	2,959 (21.3)	1,822 (18.8)	5,908 (23.7)	1,148 (9.3)	1,456 (13.3)	1,229 (12.4)	1,372 (15.6)	4,328 (19.7)
Marital status, N (%)												
Unmarried	323,649 (50.5)	284,042 (54.3)	2,551 (47.9)	37,056 (32.9)	8,755 (63.1)	4,819 (49.6)	8,891 (35.6)	2,953 (23.9)	1,851 (17.0)	3,381 (34.0)	964 (11.0)	5,442 (24.8)
Married/partner	266,481 (41.6)	200,598 (38.4)	2,395 (45.0)	63,488 (56.5)	4,503 (32.5)	4,446 (45.8)	14,140 (56.7)	7,818 (63.4)	7,452 (68.3)	4,470 (45.0)	7,020 (80.0)	13,639 (62.1)
Previously married	50,397 (7.9)	38,091 (7.3)	378 (7.1)	11,928 (10.6)	609 (4.4)	452 (4.7)	1,917 (7.7)	1,565 (12.7)	1,608 (14.7)	2,092 (21.0)	790 (9.0)	2,895 (13.2)
Motherhood, N (%)												
Yes	447,017 (69.8)	375,314 (71.8)	2,675 (50.2)	69,028 (61.4)	7,736 (55.8)	5,183 (53.3)	13,151 (52.7)	8,481 (68.8)	8,179 (75.0)	6,806 (68.5)	6,832 (77.9)	12,660 (57.6)
No	193,510 (30.2)	147,417 (28.2)	2,649 (49.8)	43,444 (38.6)	6,131 (44.2)	4,534 (46.7)	11,797 (47.3)	3,855 (31.3)	2,732 (25.0)	3,137 (31.5)	1,942 (22.1)	9,316 (42.4)

*All measurements are for the last year individuals are observed in the dataset.*

a *Except of income and income distribution which are means and medians for all year's individuals are contributing in the study*.

**Figure 1:**
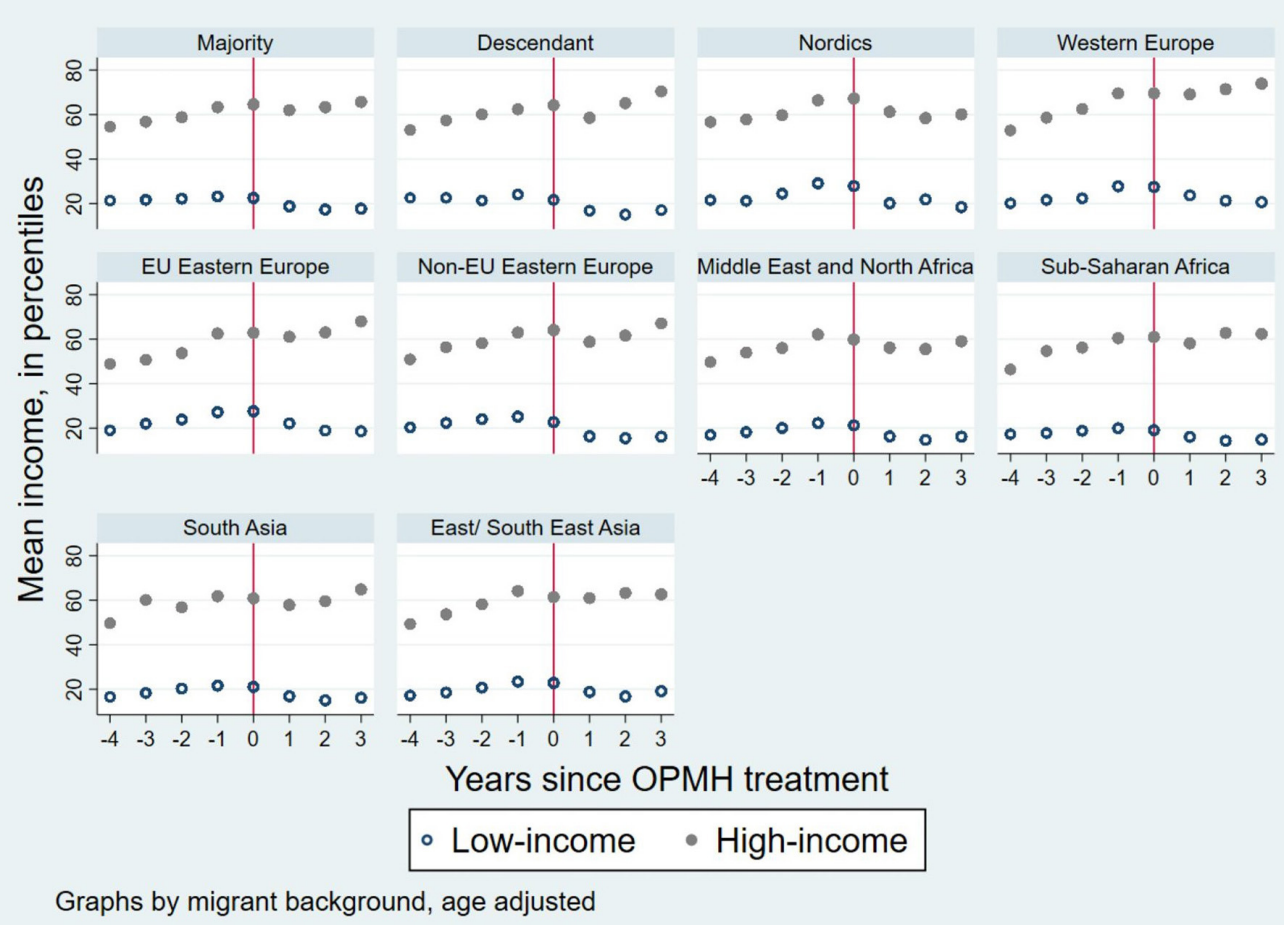
Age adjusted mean income for women with OPMH treatment in the years prior to and after treatment. Graphs by migrant background and income group.

Figure 1 presents the age-adjusted mean income in percentiles, by migrant background for women with OPMH treatment, between four years prior to, and three years after, the uptake of treatment. Low- and high-income levels are represented in the figure separately. For most groups, income flattened a year before treatment, especially among those with low income. For women in the high-income group, income tended to increase again two years after the uptake of treatment, while for women in the low-income group, it seemed to remain stable in the years following the uptake of treatment.

In order to investigate the effect of OPMH treatment on subsequent work-related income, we applied linear regression with individual fixed effects. The analysis was stratified by low- and high-income (Table 2). Results from Model 1, for both income levels, show that OPMH treatment is associated with a decrease in income. Adjustment for several covariates (Model 2) did not change the effect of OPMH treatment on the main outcome. We found that OPMH treatment had a greater adverse effect for those with low income compared to those with high income.

**Table 2:** Linear fixed effects model for the change in income^a^ following the uptake of OPMH treatment^b^.

	**Low income**						**High income**					
	**Model 1**			**Model 2**			**Model 1**			**Model 2**		
	**β**	**95% CI**		**β**	**95% CI**		**β**	**95% CI**		**β**	**95% CI**	
Uptake of OPMH treatment	−9.2	−9.4	−9.0	−9.0	−9.2	−8.8	−5.3	−5.5	−5.1	−5.3	−5.5	−5.1
Interaction between OPMH service use and migrant background												
Descendant	−2.1	−4.1	−0.1	−2.5	−4.4	−0.5	−2.2	−4.3	−0.2	−1.9	−3.9	0.1
Migrants:												
Nordics	2.0	0.6	3.4	2.0	0.6	3.4	−2.2	−3.6	−0.9	−1.1	−2.5	0.2
Western Europe	3.4	1.4	5.4	3.3	1.4	5.3	3.0	1.2	4.7	4.3	2.6	6.0
EU Eastern Europe	3.9	2.4	5.4	3.7	2.6	5.2	5.4	3.7	7.1	6.2	4.5	7.8
Non–EU Eastern Europe	−2.0	−3.3	−0.7	−1.6	−2.9	−0.3	0.8	−0.8	2.5	1.2	−0.5	2.8
MENA	1.2	0.2	2.2	1.6	0.6	2.6	−3.4	−4.9	−1.8	−3.7	−5.3	−2.2
Sub–Saharan Africa	1.9	0.4	3.5	2.1	0.6	3.7	0.3	−2.3	3.0	−0.2	−2.8	2.4
South Asia	0.8	−0.6	2.3	1.3	−0.1	2.8	−2.6	−4.9	−0.2	−2.3	−4.5	0.03
East/ South East Asia	2.4	0.9	3.9	2.6	1.1	4.1	−0.01	−2.1	2.0	−0.2	−2.2	1.9
Age	1.9	1.9	2.0	2.0	2.0	2.0	3.8	3.7	3.8	4.1	4.1	4.1
Education (ref. Compulsory or lower)												
Upper-secondary				2.5	2.2	2.8				2.9	2.5	3.2
Tertiary				8.0	7.6	8.3				19.6	19.2	20.0
Motherhood (ref. No)				−1.1	−1.3	−1.0				−7.9	−8.0	−7.8
Marital status (ref. Unmarried)												
Married/partner				1.4	1.2	1.5				0.6	0.5	0.8
Previously married				−0.7	−0.9	−0.4				1.1	0.9	1.3
Within R^2^	0.06			0.08			0.22			0.24		

a *in percentiles;*

b *for low- and high-income groups; coefficients (β) with 95% confidence intervals (95 % CI).*

*Low-income group: N = 1,348,009; n = 228,176. Obs. per group min. 3 years-max. 8 years, average 5.9 years; High-income group: N = 2,696,010; n = 412,351. Obs. per group min. 3 years-max. 8 years, average 6.5 years; Model 2 adjusted for time fixed effects*.

To investigate whether the impact of OPMH treatment on work-related income differed for the Norwegian majority, descendants and migrants, an interaction term between OPMH treatment and migrant background was introduced into the models. In the low-income group, the results in the fully adjusted model (Model 2), show that most migrant groups experience a significantly less adverse, though still negative, effect of OPMH treatment, a proxy for mental disorders, on work-related income when compared to majority women. The effect among women from South Asia, however, was not significantly different from the reference group of majority women. Another exception were women from non-EU Eastern Europe and descendant women, who had −1.6 percentile and −2.5 percentile of additional loss in income when compared to majority women. In the high-income group, only women from Western Europe and EU Eastern Europe were significantly less affected following the uptake of OPMH treatment when compared to majority women. Migrant women from MENA experienced an additional loss in income when compared to the majority. Despite the greater loss of income among women from Nordic countries, Sub-Saharan Africa, South Asia and East/South East Asia, and a smaller loss of income among women from non-EU Eastern Europe, none of these groups differed significantly from the majority women.

**Figure 2:**
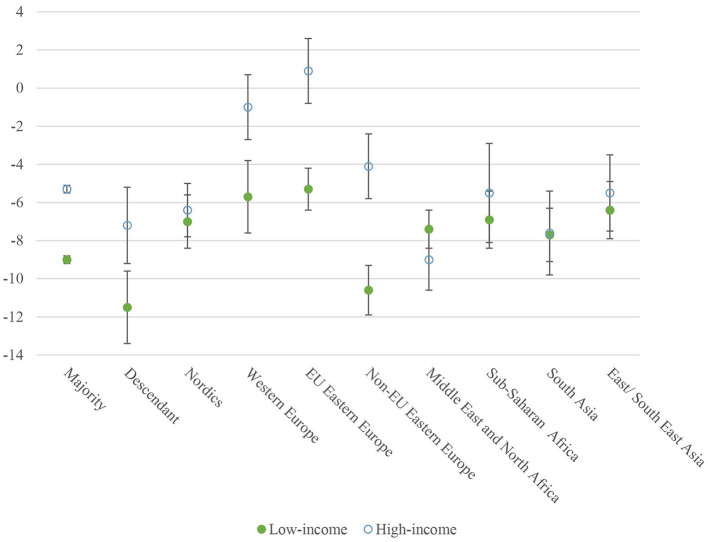
The interaction effect of OPMH service use on income by region of origin and income distribution. Adjusted for age, educational level, marital status, motherhood, and time fixed effects.

Figure 2 is a graphical presentation of the results from Table 2, Model 2, showing the adjusted effects of OPMH treatment on income by migrant background (on the x-axis) and income group. The y-axis shows the percentile loss of income. For most groups, the effect is most adverse in the low-income group, except for women from MENA. Furthermore, the change in income was negative for all groups, except for EU Eastern European women with high income, who did not experience a significant change in income following the uptake of OPMH treatment.

## Discussion

By using longitudinal register data, this study explored the changes in income following the uptake of OPMH treatment, a proxy for mental disorder, by migrant background and income level among women aged 23–40 years in Norway. To the best of our knowledge, this is the first study to examine the effect of OPMH treatment on income in migrant-, descendant- and majority women. We found that OPMH treatment had a significantly negative impact on work-related income, which supports the social selection perspective. Adjustment for several time-varying background variables such as educational level, marital status and motherhood did not alter this. Our results are in accordance with previous research showing that mental disorders are associated with subsequent income loss (36–39). Furthermore, we also found the effect of mental disorders, defined by OPMH treatment, to vary by income level, with greater adverse effects among women in the low-income group. This finding confirms previous research (38, 39). However, our hypothesis regarding a greater loss of income among migrant women, when compared to majority and descendant women, was mostly rejected. We found that the change in income following the uptake of OPMH treatment was negative for all groups, except for Western and EU Eastern European women in the high-income group who did not actually experience a significant change in income. Only descendant and migrant women from non-EU Eastern Europe with low income, and women from MENA with high income, experienced a greater loss of income when compared to majority women. All the other groups either did not differ significantly from majority women or experienced a smaller loss in income following the uptake of OPMH treatment. When accounting for several background variables, only a minor part of the income loss following the uptake of OPMH treatment was explained. However, education level, motherhood and marital status explained the difference in income loss between majority and descendant women and migrant women from Nordic countries and South Asia with a high income.

There are several potential explanations for the larger loss of income among users of OPMH with low income. One potential reason for greater income loss among women with low income could be that women with low income experience more severe disorders compared to women with high income. Luciano and Meara (47) found that the proportion of unemployed and individuals with low income increased with severity of mental disorder. However, this explanation cannot be confirmed or rejected by the data used in this study. Future research should address this issue and studies should include the severity of a mental disorder (e.g., OPMH service use vs. hospitalization) when investigating the association between mental disorder and income or other work-related outcomes. Furthermore, women with low income may suffer greater loss of income following mental disorder as they are more likely than those with high income to experience financial difficulties which can again affect recovery. For some groups of women, the income loss following the uptake of OPMH treatment seems to be temporary, while for others the effect may be more long-lasting, as indicated in Figure 1. Another potential explanation is that the larger loss of income in the low-income group may be a result of poorer labor market attachment among these women, e.g., they could work part-time or work on short-term contracts. Additionally, women in the high income group may have better coping and negotiation skills that allow them to function in their work roles better, even if they have a mental disorder. They may be more able to negotiate a temporary deal to combine their illness and work with their employer or have a more flexible type of job.

However, the above suggested interpretations may not necessarily apply to migrant women. Despite the higher likelihood of migrant women being in precarious employment and low-skilled jobs than the majority population (22), mental disorders seem to have a less adverse, though still negative, effect on their income compared to majority women. This may be a result of postponement of help seeking among some migrant women, especially those with low income. Thus, the impact of mental disorder on income may have occurred already prior to help seeking. Further, in Norway, the social benefit is calculated based on the income from the last few years prior to becoming ill or injured (48). Thus, as many migrant women have more precarious labor market attachment, it is possible that the financial compensation that migrants get when they become ill or lose their job is lower than that for the majority group. Thus, many may be motivated to stay at work while ill, despite reduced productivity.

Among those with high income, migrant women from the three European groups experienced a smaller income reduction compared to the majority. Women from Western and EU Eastern Europe, in both income groups, are largely labor migrants (25), who may only have short-term plans of remaining in the country. Thus, their motivation for staying in work despite mental health difficulties can result in a smaller income reduction than for other groups. In addition, these European women may have better protection than some other migrant groups because they are more likely to enter the Norwegian labor market with valuable skills that are in demand. On the other hand, it is possible that Western and EU Eastern European migrant women who attend mental healthcare services are less unwell than majority women, descendants, and other investigated migrant groups. However, OPMH services are usually reserved for individuals with severe and enduring disorders. Most mild to moderate mental health disorders are treated at the primary care level (49). However, as previously mentioned, this study was not able to investigate the severity of mental disorders that women seek help for, thus this hypothesis could not be further investigated.

The larger income loss due to mental disorders, measured by OPMH treatment, among migrant women from MENA and South Asia with high income compared to majority women, could also be a result of a higher share of these women being in precarious employment (such as short-term contracts) (22). Compared to European migrants, women from MENA and South Asia are less likely to enter Norway as labor migrants, thus they appear more likely to fall out of the labor market when their work ability and productivity declines. Thus, income in these migrant groups may decrease more than for labor migrants or majority women. Helgesson et al. (41) found that labor market marginalization of migrants can mainly be explained by poor labor market attachment of migrants prior to experiencing a mental disorder when compared to non-migrants. Authors also found migrants experiencing mental disorders were at increased risk of unemployment. The type of mental disorder individuals in the studied groups are treated for could also explain some of the differences we see in income loss. Studies investigating the association between mental disorders and income found for instance, that the effect was stronger for anxiety disorders than for personality disorders or dysthymia (38). Previous research found migrant women, mainly from low-income countries, had higher levels of common mental disorders such as anxiety (10). Thus, selection of migrant women with, for instance, anxiety into the high-income group could explain the larger loss of income among non-Western migrant women. However, in this study, we were unable to investigate the differences by the type of mental disorder.

In order to prevent or reduce the income loss due to mental disorders in affected women, we suggest several structural changes. Demands for employers to offer a higher share of permanent employment as well as more flexibility and adjustment of tasks could result in a higher probability of women remaining in the labor market after the uptake of OPMH treatment. However, we are aware of that this may be challenging particularly given the Covid-19 pandemic that has affected the hotel- and restaurant sector, retail, and transportation in particular. Migrants to a greater extent work in these sectors and positions and have thus been more affected by restrictions (50). Furthermore, the pandemic has highlighted their vulnerable and precarious position in the labor market even more (50, 51). Thus, strategies to offer more permanent contracts should be pursued post-pandemic. Moreover, according to the Norwegian Labor Inspection Authority, employers have a responsibility to follow up their employees, and particularly those with reduced workability due to, for instance, mental disorder. In collaboration with the employee, they must consider implementing measures that can help the employee to return to work following sick leave. Examples of measures can be adjustment of existing tasks or agreement about alternative working tasks, adjustment of working hours and the working environment that can help an employee on a sick leave to return to work either part-time or full time (52). Furthermore, Norwegian Labor and Welfare Administration (NAV), provides follow up and supervision for individuals with mental health problems and those in treatment for mental disorders, including substance abuse, to help them to stay or return to work (53). A report on collaboration between OPMH services and NAV, has shown increased collaboration between these two institutions and the use of work-related activities as a part of treatment of individuals with mental disorders (54). However, the extent to which the implementation of measures to help an employee to return or stay in work despite mental disorder is practiced, especially among the precariously employed, and whether any interventions are targeted toward migrants with mental disorders in particular, is rather unclear. As labor market participation is important for both social and economic integration of migrants (22) and for mental health, a particular focus should be on keeping migrant women in employment. Therefore, we encourage future interventions and policies to focus their attention on migrants with mental disorders, and their needs to stay in, or enter, the labor market. Additionally, contact with healthcare services should be more effective and adjusted to the needs of both migrant and non-migrant women. As migrant background can potentially be a risk factor for not seeking treatment (18), services that improve access to the healthcare system could potentially result in timely and appropriate use of mental health care services. When in treatment, consideration of the differences between different migrant groups with regard to their reasons of migration (25), their level of educational attainment (26) and the type of work they are performing (50), but also their understanding of mental health (55) and type of disorders they develop (13, 14, 16, 17) are all important to take into account when planning a successful treatment targeted toward affected individuals. Thus, mental healthcare services culturally and linguistically adapted to the needs of migrants could increase healthcare use of this group (56). Finally, policies around sick leave and income support may be important for ameliorating the impact of mental disorders on one's income.

### Strengths and Limitations

This study has several strengths. Use of a dynamic sample allowed us to follow women entering the study sample later than at the study start in 2006, due to immigration or reaching the adequate age, and to include women who died or emigrated during the study period as long as they had at least three consecutive years in the study. Furthermore, we only included potentially healthy women, since we applied a two-year wash-out period to ensure that we measured the change in income due to use of such services. However, to access OPMH services, the referral from a general practitioner or psychologist is required and it may take a long time before an appointment is made. Thus, even before entering OPMH treatment, individuals may have been affected by mental disorders for a significant amount of time and their income may have already started to decrease or stagnate by the time we considered them as exposed, as suggested by Figure 1.

By using information on OPMH treatment from national registers, we rule out any self-reported bias. Migrants might not only participate in surveys to a lesser extent due to language difficulties but also, when participating, give biased answers due to the experienced discrimination and negative perceptions about migrants (57). However, use of OPMH services only detects those who entered the services and not all women experiencing mental disorders for instance those only consulting their general practitioner. Migrants may face a range of barriers when seeking mental healthcare (58), including culturally rooted stigma toward seeking mental healthcare, linguistic barriers, and limited knowledge about the healthcare system (59, 60). There are several steps that need to be undertaken before entering OPMH services and individuals in OPMH treatment need to have actively sought help (61).Therefore use of OPMH treatment as a proxy for mental disorder may be a poorer measure among migrants than among majority women. Furthermore, we also lack information on those using private healthcare services or inpatient services. However, inpatient treatment accounts for only about five percent of all contacts with mental healthcare services (62). The operationalization of mental disorder is therefore the main limitation of this study. Additionally, previous research suggested that for individuals with severe mental disorders such as schizophrenia, labor market participation is limited. In many cases, these individuals never enter the labor market (35). Thus, since we excluded individuals with no income during the study period, we may have excluded the most marginalized women in our study.

Lastly, it is important to stress that information contained in registers is not collected for research purposes. We are therefore restricted to using available variables, pre-defined categories and the time frame for when the information was collected (63).

Despite these limitations, utilization of a more detailed grouping of migrants is novel with regard to studying this association. Further, use of register data to gain information on income is an advantage due to no missing income data, as all individuals with paid employment in Norway are registered in the database. Self-reported income is a sensitive topic, and often suffers from large numbers of missing responses (64). Whether our data can be generalized to other countries needs to be examined in future research. However, we feel that this might be the case and that our results potentially have implications for other welfare states. As stated in the introduction section, women, and migrant women in particular, do not only occupy a disadvantaged position in the labor market in Norway, but also in other European countries (22, 23). Thus, it is reasonable to assume that loss of income due to mental disorder, and the differences between majority and migrant women may apply also in other countries with similar employee protection laws.

## Conclusion

In summary, this study improves our understanding of the association between OPMH treatment and subsequent work-related income among migrant and non-migrant women in Norway. We found OPMH treatment resulted in loss of income among women, irrespective of migrant background or initial income level. However, our study also found that the magnitude of income loss was larger for women with low income and varied by migrant background. In our analyses, we controlled for factors such as educational level, marital status and motherhood in addition to time fixed effects, and our fixed effects approach controlled for all unmeasured time-invariant confounders. Even with these controls, the main effect of OPMH treatment on income remained virtually unchanged. Therefore, in this study, the income loss following the uptake of OPMH treatment cannot be explained by sociodemographic factors. Although possible explanations of this relationship have been discussed, more research investigating the mechanisms involved is needed. Many women, during early adulthood, are starting their career and its disruption can strongly influence their future employment trajectories or their long-term dependency on welfare services. This can in turn, affect future mental health, resulting in a vicious circle and contributing to more inequalities. This may result in large costs for the society at large.

## Data Availability Statement

The datasets generated and analyzed for the current study are not publicly available for data protection reasons. However, the data that support the findings of this study may be available from Statistics Norway and HELFO if ethical approval is granted. Requests to access these datasets should be directed to Statistics Norway (https://www.ssb.no/en/data-til-forskning/utlan-av-data-til-forskere) and HELFO (https://www.helsedirektoratet.no/tema/statistikk-registre-og-rapporter/helsedata-og-helseregistre/kuhr#sokomdatafrakuhr).

## Ethics Statement

The studies involving human participants were reviewed and approved by the Regional Committee for Medical and Health Research Ethics, South East Norway (REK 2014/1970) and the owners of the different registries approved the linkage of data. Written informed consent for participation was not required for this study in accordance with the national legislation and the institutional requirements.

## Author Contributions

KH designed the study, carried out the statistical analysis, interpreted the data, and drafted the manuscript under supervision. A-CH and AL contributed critically to the design of the study and to revising the manuscript. LH co-supervisor, contributed to the design of the study, prepared the data file, and contributed to revising the manuscript. MS main supervisor, interpreted the results, contributed critically to the design of the study, and to revising the manuscript. All authors approved the final version of the manuscript.

## Funding

This research was funded by the Research Council of Norway through the Women's Health programme' (grant number: 273262/H10). A-CH's contribution to the article was funded by Hollander/Forte 2016-00870/Psykiatrisk vård bland utrikesfödda.

## Conflict of Interest

The authors declare that the research was conducted in the absence of any commercial or financial relationships that could be construed as a potential conflict of interest.

## Publisher's Note

All claims expressed in this article are solely those of the authors and do not necessarily represent those of their affiliated organizations, or those of the publisher, the editors and the reviewers. Any product that may be evaluated in this article, or claim that may be made by its manufacturer, is not guaranteed or endorsed by the publisher.
